# Dynamic light scattering studies of the effects of salts on the diffusivity of cationic and anionic cavitands

**DOI:** 10.3762/bjoc.14.195

**Published:** 2018-08-23

**Authors:** Anthony Wishard, Bruce C Gibb

**Affiliations:** 1 Department of Chemistry, Tulane University, New Orleans, LA 70118, USA

**Keywords:** cavitand, dynamic light scattering, Hofmeister effect, ion–ion interactions, water

## Abstract

Although alkali halide salts play key roles in all living systems, the physical models used to describe the properties of aqueous solutions of salts do not take into account specific ion–ion interactions. To identify specific ion–ion interactions possibly contributing to the aggregation of proteins, we have used dynamic light scattering (DLS) to probe the aggregation of charged cavitands. DLS measurements of negatively charged **1** in the presence of a range of alkali metal halides reveal no significant aggregation of host **1** as a function of the nature of the cation of the added salt. Only at high concentrations could trace amounts of aggregation be detected by ^1^H NMR spectroscopy. Contrarily, **1** was readily aggregated and precipitated by ZnCl_2_. In contrast, although fluoride and chloride did not induce aggregation of positively charged host **2**, this cavitand exhibited marked aggregation as a function of bromide and iodide concentration. Specifically, bromide induced small but significant amounts of dimerization, whilst iodide induced extreme aggregation. Moreover, in these cases aggregation of host **2** also exhibited a cationic dependence, with an observed trend Na^+^ > Li^+^ > K^+^ ≈ Cs^+^. In combination, these results reveal new details of specific ion pairings in aqueous solution and how this can influence the properties of dissolved organics.

## Introduction

Although all life on planet Earth depends on aqueous solutions, our understanding of aqueous supramolecular chemistry is limited. As a result, as Smith has eloquently pointed out [1], the effects of buffers and salts on dissolved organics can be quite bewildering. Why is this? The proverbial elephant in the room is that classical theories of electrolytes rest on the assumptions that all ions are point charges that only form non-specific interactions. The ramifications of this are innumerable. For example, pH measurements are based on extended Debye–Hückel theory [2] and Poisson–Boltzmann distribution [3] to describe ionic profiles near the glass-electrode surface. These classical models may be good approximations for ions such as Li^+^ and F^−^, but they are poor models for ions that don’t behave as hard point charges [4]. Correspondingly, IUPAC advises researchers to avoid pH measurements above 0.1 M to minimize errors [5]. Related problems lie with Derjaguin, Landau, Verwey and Overbeek (DLVO) theory as a model of the aggregation of aqueous dispersions. DLVO often quantitatively succeeds, but it fails to predict ion specific effects [4,6–7]. Similarly, it is becoming increasingly evident that the Hofmeister and reverse Hofmeister effects [8–9] – most commonly discussed in terms of how salts affect biomacromolecules – can only be fully understood in terms of specific ion–ion, ion–water, and/or ion–macromolecule interactions [4,10–11].

Although many attempts have been made to amend these and other classical models [4], success has been limited because of our lack of understanding of the specific supramolecular properties of individual ions. There is therefore an opportunity for supramolecular chemists (who by their very training demand specificity of interactions) to help build a full understanding of ion-specific interactions in water and help usher the troubling elephant out of the room.

Recently we demonstrated how host molecules can engender the Hofmeister [12–13] and the reverse Hofmeister effects [14]. In regards to the former, we have shown how poorly solvated anions such as SCN^−^ have an affinity for non-polar surfaces. Because of this, they can compete with the interactions between two non-polar surfaces in a host–guest complexation event and can induce an apparent weakening of the hydrophobic effect akin to how these anions can partially unfold proteins. Alternatively, poorly solvated anions can also associate closely with cationic groups, induce charge neutralization, and engender aggregation and/or precipitation. In other words, they can also cause an apparent increase in the hydrophobic effect. This is the reverse Hofmeister effect, and in complex biomacromolecules we surmise that both effects are in operation, and that in very general terms it is the balance between these that dictates the properties of a particular macromolecule under specific conditions. Cations can also induce Hofmeister effects, but these are usually much weaker, and we believe there are two reasons for this. First, simple metal cations are generally more strongly solvated than comparable anions that can induce Hofmeister effects. Second, the anions that predominate in biomacromolecules are carboxylates, phosphates and sulfates, and the strong solvation of these means that it is hard for a cation to form an ion pair and induce Hofmeister effects.

**Figure 1:**
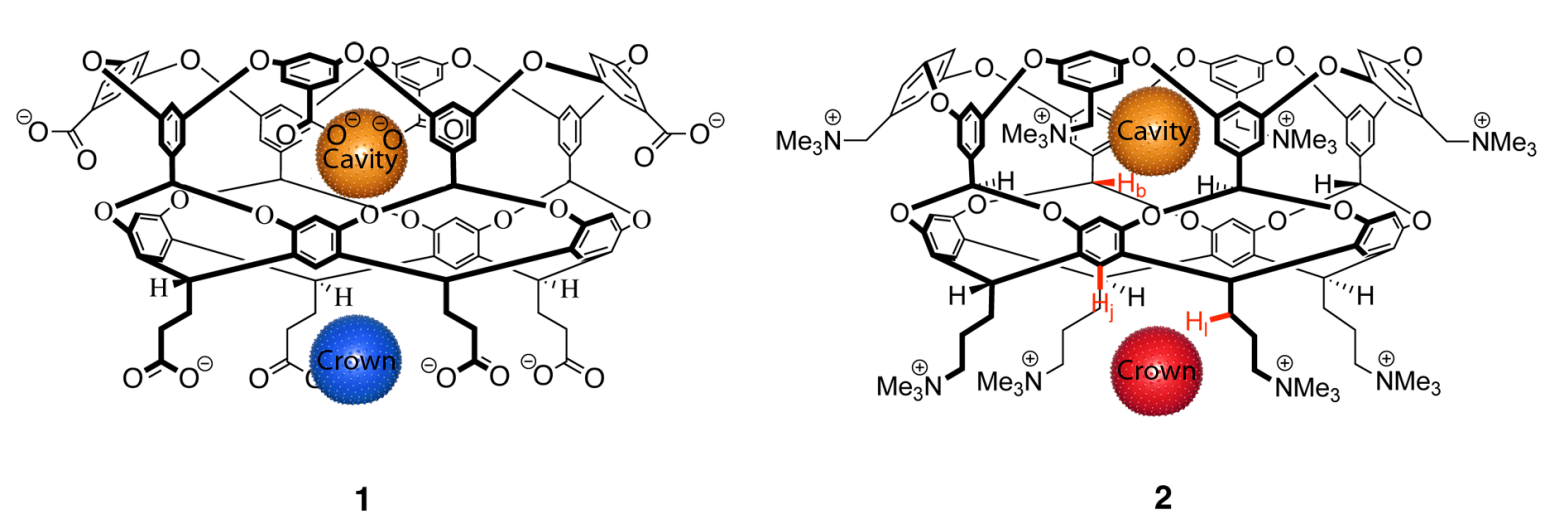
Chemical structures of octaacid **1** and positand **2** showing the anionic binding sites of the two hosts (orange and red) and the potential cationic binding site of **1** (blue).

To explore these ideas further we report here the responses of two deep-cavity cavitands, octacarboxylate **1** (counter ion Na^+^) [15–16] and positand **2** (counter ion Cl^−^) [14] (Figure 1), to different salts using dynamic light scattering (DLS) [17–20]. Respectively functionalized with carboxylates and trimethylammonium groups, these hosts are expected to possess unique ion-pairing properties and hence have very different reverse-Hofmeister responses to added salts. More specifically, both octacarboxylate **1** and positand **2** have a non-polar cavity that can function as an anion (but not to our knowledge a cation) binding site. Anion binding to the cavity of positively charged **2** is stronger than to negatively charged **1** [13–14], but nevertheless anion binding to **1** can be as strong as 4.60 kcal mol^−1^. Host **2** has a second anion binding site in the form of the crown of trimethylammoniums “under” the primary bowl [14], and correspondingly the four chelating carboxylates of the crown of **1** may be a reasonable cation binding site. Furthermore, in addition to these specific cavity and crown sites, the individual charge groups of **1** and **2** can function as weak (pseudo-specific) binding sites for ions of opposite charge.

## Results and Discussion

**Figure 2:**
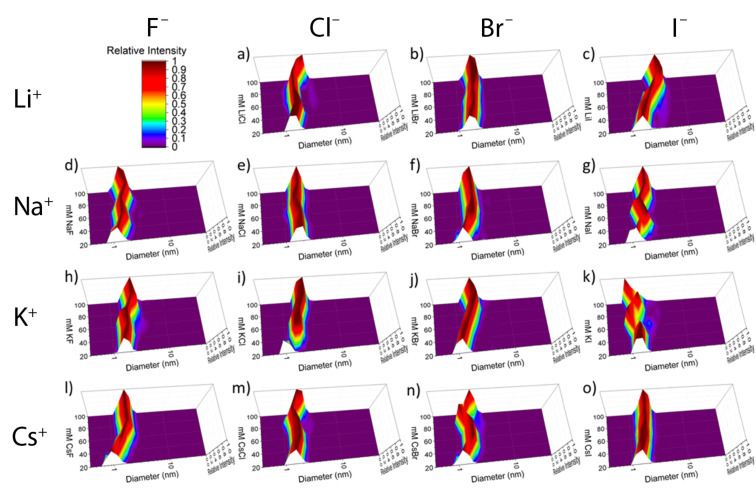
Representative plots of the volume-weighted distribution obtained by DLS for salts titrated into 2.00 mM **1**: a) LiCl, b) LiBr, c) LiI, d) NaF, e) NaCl, f) NaBr, g) NaI, h) KF, i) KCl, j) KBr, k) KI, l) CsF, m) CsCl, n) CsBr, o) CsI. Scale shown in the upper-left corner. The *x*-axis represents hydrodynamic diameter, the *y*-axis the concentration of the respective salt (mM), and the *z*-axis the relative intensity.

To determine the effects of salts on **1** (counter ion Na^+^) DLS was used to monitor its observed hydrodynamic volume during titration with various halide salts. The fifteen salts studied were a matrix of the alkali metal cations Li^+^, Na^+^, K^+^, and Cs^+^ in combination with the halides F^−^ through I^−^, the one omission being poorly soluble lithium fluoride (maximum solubility = 0.134 g mL^−1^). Unsurprisingly, given the p*K*_a_ values of carboxylic acids, host **1** has limited solubility in unbuffered water. Thus for solubility reasons, titrations of **1** were performed in 20 mM NaOH solution (see Supporting Information File 1, Figure S2, for more details). In each case titrations were taken to 100 mM salt where it was assumed that the host is fully screened [21–23]. Figure 2 shows the effects of the different salts on the observed size of **1**.

The reported hydrodynamic diameters were calculated using the Stokes–Einstein equation (Equation 1), which assumes host **1** is a spherical particle,

[1]
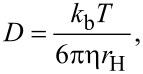


where *D* is the diffusion constant, *k*_b_ is the Boltzmann constant, *T* is the temperature, η is the viscosity of the solution, and *r*_H_ is the hydrodynamic radius.

In all cases, at the initial 20 mM concentration of NaOH the light scattering induced by **1** was weak. This resulted in relatively flat autocorrelation functions generated from the measured fluctuations in scattered light. Consequently, the recorded size of the host was both anomalously small and highly variable, covering the range 1.0 to 1.7 nm (Figure 2). This compares to molecular models which show host **1** approximates to an anti-cube (square antiprism) with sides of ≈ 2.0 nm. The weak light scattering of **1** was attributed to the high charge density of the host and the low ionic strength of the solution engendering significant Coulombic interactions between host molecules [24]. Titrating samples with the different salts led to much stronger light scattering and an apparent increase in the hydrodynamic diameter of the host to a more realistic ≈ 2 nm. In all cases, however, the nature of the cation had no perceivable effect; each metal ion resulted in a hydrodynamic diameter for **1** of 2.1 ± 0.2 nm (Table 1). The invariance in these results reveals the power of the carboxylate as a water-solubilizing group. Although its p*K*_a_ may not be optimal for deprotonation at neutral or physiological pH, its small size and relatively high free energy of hydration (−373 kJ mol^−1^) ensure that ion-pairing effects are not strong. This was further confirmed by ^1^H NMR spectroscopy (Supporting Information File 1, Figure S3), which revealed only trace amounts of host aggregation (≈5%). Furthermore, even at 100 mM salt concentration, ^1^H NMR spectroscopy failed to show any significant association of Cs^+^ to the crown of four carboxylates. Thus, although this crown is the most obvious potential cation binding site, we see no evidence of specific complexation here. More generally, despite the high charge density of **1**, monovalent alkali metal ions cannot associate with it sufficiently to induce significant aggregation and a reverse Hofmeister effect. This was not, however, the case with divalent metal ions, which are well recognized to interact strongly with carboxylates and induce aggregation [25]. Thus, visual inspection upon the addition of ZnCl_2_ to give a 100 mM salt concentration revealed extensive precipitation of the host. Returning to the point that the majority of anionic groups in biomacromolecules are strongly solvated, it is interesting to contemplate the idea that the prevalence of alkali metal ions in the environment exerted evolutionary pressures on biomacromolecules to select carboxylate, phosphates and sulfates and hence minimize ion pairing, charge neutralization, and deleterious precipitation effects in living systems.

**Table 1:** Summary of titration data from DLS experiments.

cation	anion	host **1**^a,b^ max. dia. (nm)	host **2**^a,b^ max. dia. (nm)	*n*-mer aggregate^b^ # (for host **2**)

Li^+^	F^−^	–^c^	–^c^	
	Cl^−^	2.1 ± 0.0	2.1 ± 0.1	
	Br^−^	2.1 ± 0.2	2.5 ± 0.0	1.7 ± 0.0
	I^−^	2.0 ± 0.0	14.3 ± 0.5^d^	314 ± 32^d^

Na^+^	F^−^	1.8 ± 0.1	1.8 ± 0.3	
	Cl^−^	2.0 ± 0.2	2.1 ± 0.1	
	Br^−^	1.8 ± 0.0	2.6 ± 0.1	1.8 ± 0.1
	I^−^	2.0 ± 0.1	18.6 ± 3.1^d^	764 ± 260^d^

K^+^	F^−^	2.1 ± 0.4	2.0 ± 0.1	
	Cl^−^	2.1 ± 0.1	2.0 ± 0.0	
	Br^−^	2.1 ± 0.1	2.4 ± 0.1	1.4 ± 0.1
	I^−^	2.2 ± 0.3	11.6 ± 0.8	170 ± 37

Cs^+^	F^−^	1.9 ± 0.0	2.0 ± 0.3	
	Cl^−^	2.0 ± 0.1	2.0 ± 0.0	
	Br^−^	1.9 ± 0.1	2.4 ± 0.0	1.5 ± 0.0
	I^−^	1.9 ± 0.1	11.9 ± 0.2	180 ± 10

^a^Determination of the maximum hydrodynamic diameter (max. dia.) was made regardless of the salt concentration at which the maximum size occurred. In the event of a bimodal distribution, the mode that accounted for >10% of the total distribution and had the largest diameter was used to determine max. dia. ^b^Values are the average of two datasets. ^c^For solubility reasons titrations with LiF were not performed. ^d^Values are the average of three or more datasets.

**Figure 3:**
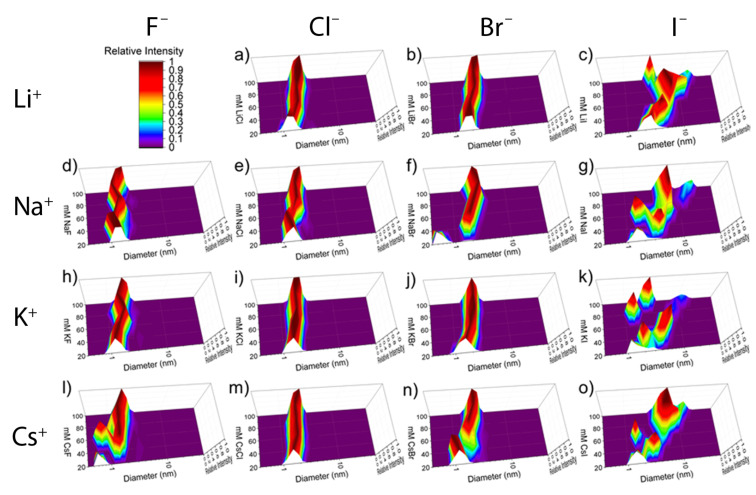
Representative plots of the volume-weighted distribution obtained by DLS for salts titrated into 2.00 mM **2**: a) LiCl, b) LiBr, c) LiI, d) NaF, e) NaCl, f) NaBr, g) NaI, h) KF, i) KCl, j) KBr, k) KI, l) CsF, m) CsCl, n) CsBr, o) CsI. Scale shown in the upper left corner. The *x*-axis represents hydrodynamic diameter, the *y*-axis the concentration of the respective salt (mM), and the *z*-axis the relative intensity.

Overall, octacarboxylate **1** is a binder of large, polarizable anions in its non-polar pocket [12], but is not a perceptible binder of alkali metal cations. Building on this, we carried out similar DLS studies with host **2** (counter ion Cl^−^) using the same aforementioned salts (Figure 3). In a previous work, our DLS studies of this host involved solutions buffered with 40 mM phosphate (pH 7.3) [14]. Under these conditions, the initially measured sizes in the absence of added salt were consistently 1.9–2.1 nm; values that match the modeling of the host. In stark contrast to this earlier work, but analogously to host **1**, when we examined solutions of **2** in the absence of any added buffer and salt, light scattering was weak. This resulted in flat autocorrelation functions and again an anomalously small and highly variable hydrodynamic volume (0.5–1.4 nm). This issue noted, at the titration point of 80 mM salt the curvature of the autocorrelation function greatly increased, and the observed hydrodynamic diameter approached the expected ≈2.0 nm. Hence although for all of the studies here the starting point for each titration was 20 mM salt, the first data point for each titration was ignored.

An obvious trend in the data for host **2** (Figure 3) is how the nature of the halide affects aggregation. Over all concentrations of F^−^ salts the hydrodynamic diameter of the host was anomalously small, with maximum diameters measured in the presence of NaF, KF, and CsF being 1.6, 1.9, and 1.8 nm. The strongly solvated F^−^ ion [26] has a very weak affinity for host **2** [14], and we therefore interpret these small hydrodynamic diameters to limited binding to host **2** and hence an inability to screen interhost interactions. In contrast, in the presence of at least 80 mM Cl^−^ salts the hydrodynamic diameter of host **2** was consistently within the expected range of 2.0–2.1 nm. Thus, independent of the metal cation Cl^−^ is an ideal anion for effectively screening intermolecular charge–charge interactions between the host (Table 1). The case of Br^−^ was quite different. For all salts, the addition of Br^−^ leads to an increase in the hydrodynamic diameter, with NaBr giving the largest increase to 2.6 nm. This corresponds to the formation of a dimer aggregate. Aggregation was even more extreme with the I^−^ salts. All I^−^ salts caused extensive aggregation of the host, and a determination of the maximum size induced by the four salts ranged from 170 and 180-mers in the presence of KI and CsI, to ≈724-mers for NaI. Evidently the difference in the free energies of solvation of Br^−^ and I^−^ (Δ*G*_hyd_ = −321 and −283 kJ mol^−1^, respectively) is key to allowing more ion pairing between the trimethylammonium groups of **2** and I^−^ to induce substantial aggregation.

**Scheme 1:**
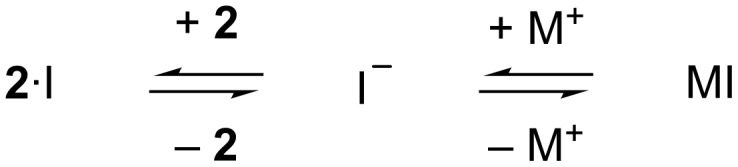
Visualization of the competitive equilibrium between iodide binding to host **2** or associating with its alkali metal cation M^+^. Non-competing Cl^−^ is omitted for simplicity.

The data for the I^−^ salts illustrate a further complexity to the ability of salts to induce precipitation of ammonium ions such as **2**. Thus, although the aggregation induced by KI and CsI are not significantly different, there is a trend for cation-induced aggregation of cationic **2**: namely Na^+^ > Li^+^ > K^+^ ≈ Cs^+^. This cation effect must be indirect. If the counter ions of **2** (Cl^−^) are viewed as non-coordinating, this phenomenon can be interpreted as arising from a simple competition between the two “hosts” **2** and M^+^ (Scheme 1). In such a system, I^−^ can only associate with host **2** and induce charge neutralization and aggregation when it is in the free state, but if it itself strongly associates with the counter ion of the salt then it will not be able to bind strongly to **2**.

There is a well-established “volcano plot” relationship between the standard heat of solution of crystalline alkali halides and the difference between the absolute free energy (or heat) of hydration of the corresponding anion and cation [27–28]. As a result, in the words of Fajans, “in the case of alkali halides, the solubility in a number of salts with the same cation (anion) and different anions (cations) is at a minimum when the cation and anion are approximately equal and increases with increasing difference of the ionic radii” [29]. This has been built upon by Collins who proposed that the difference in heats of hydration of a cation and anion is a surrogate for the extent of anion pairing in solution; that small (large) anions preferentially bind with small (large) cations, whereas large size differences lead to weak association [30]. Thus, this law of matching water affinities (LMWA) suggests that in aqueous solution CsI is more strongly ion-paired than LiI. The observed trend in the aggregation of **2** (NaI > LiI > KI ≈ CsI) is therefore not in full agreement with the LMWA. The LMWA correlates with our data that K^+^ and Cs^+^ should pair strongly with I^–^ and therefore induce weak aggregation. However, it also predicts that Li^+^ and I^−^ should form the weakest ion pair and that therefore LiI should be the greatest aggregator of **2**. As Table 1 reveals, this is not the case; it is NaI that has the strongest influence on the host.

A straightforward answer for this may be that entropy is a part of the aggregation of **2**, whereas the LMWA is purely enthalpically based. Additionally, however, the absolute heats of hydration of anions and cations calculated by Morris make many assumptions, and in part rely on models that assume ideality for their determinations.

Cation effects for the bromide salts are less pronounced than those of the iodide salts, nevertheless there are small but significant differences between the pairs of cations Li^+^/Na^+^ and K^+^/Cs^+^. The former pair, as would be expected considering the data for iodide salts, leads to greater aggregation than that observed with the potassium and cesium salts. These results reveal that in contrast to host **1**, the weakly solvated groups of **2** result in significant ion-pairing effects that can, in extreme cases such as I^−^ salts, lead to a pronounced reverse Hofmeister effect. Importantly, our DLS studies reveal that this effect is also influenced indirectly by the nature of the cation of the salt.

## Conclusion

Dynamic light scattering reveals the ion-specific interactions of carboxylate and trimethylammonium groups, and hence the inherent asymmetry between negatively and positively charged molecules. The negatively charged solubilizing groups of host **1** are relatively strongly solvated, so much so that the nature of the alkali metal cation has very little effect on aggregation. Divalent metal ions are required to induce aggregation in this host. In contrast, the more weakly solvated charged groups of **2** allow ion-specific interactions with halide anions. Specifically, weakly solvated I^−^, and to a lesser extent Br^−^, can associate closely with host **2**, induce charge neutralization, and hence bring about aggregation. Importantly, because of the power of I^−^ to induce aggregation in **2** it is even possible to observe how ion pairing within a salt can influence its aggregation ability. Considering the ubiquity of alkali metal halide ions in Nature, we are examining other systems to provide greater detailing of how the balance of ion pairing in two-cation/two-anion systems influences Hofmeister effects.

## Experimental

Reagents were purchased from the commercial supplier Sigma-Aldrich Corp. and were used without further purification. Deuterated solvents were purchased from Cambridge Isotopes and used without further purification. Hosts **1** and **2** were synthesized by the procedures reported previously [15–16,31]. All ^1^H NMR spectra were collected on a Bruker 500 MHz spectrometer at 25 °C. Spectral processing was performed using Mnova software (Mestrelab Research, S.L.). All dynamic light scattering measurements were performed on a Nicomp ZLS Z3000 particle size analyzer (Particle Sizing Systems – Port Richey, FL), with a 50 mW laser diode (660 nm wavelength) and an avalanche photodiode (APD) detector. Measurements of scattered light were made at 90°, with data collected at 23 °C and processed using a non-negative least squares Nicomp analysis.

### DLS solution preparation and analysis procedures

All solutions of **1** were prepared in 20.0 mM NaOH in 18.2 MΩ·cm Milli-Q H_2_O. Solutions of **2** were prepared in unbuffered 18.2 MΩ·cm Milli-Q H_2_O. All host solutions were prepared at a concentration of 2.00 mM. Solutions of **1** and **2** were titrated with a 2.00 M salt solution in aliquots of 20 mM until reaching a final concentration of 100 mM (50 equiv) salt. Dilution of the host solution during the titration was maintained at <5% for all titrations.

Samples were centrifuged for 10 min at 10,000 rpm prior to each titration but not centrifuged thereafter. Solutions of host were titrated with salt, then shaken and vortexed to ensure mixing before acquiring DLS measurements. For each data point in a titration, analyses were performed in quadruplicate at a channel width of 5 µs. Particularly, at low salt concentrations, weak light scattering resulted in a flat autocorrelation curve; this data was immediately discarded. Of the remaining data, that with the lowest fit error was kept. At every salt concentration, the data was replicated a minimum of one time using a separate solution of host. Those data were then averaged and presented herein. Results shown are representative of the volume-weighted distribution. Surface plots of the raw, volume-weighted distribution data were plotted using OriginPro software.

### NMR solution sample preparation and analysis procedures

Monodispersity of the host **1** solution was confirmed by Pulsed Gradient Spin Echo (PGSE) NMR (Supporting Information File 1, Figure S1) in H_2_O locked with D_2_O in a 5 mm coaxial capillary insert (Wilmad-Labglass – Vineland, NJ). The concentration of the stock solid was determined by titration in triplicate with a 25.0 mM sodium ethanesulfonate (SES) solution, and integration of the methyl or methylene peaks of ethanesulfonate and the H_l_ peak of the host.

Solutions for NMR titrations were prepared in 13.0 mM NaOH in D_2_O (Supporting Information File 1, Figure S2). Titrations of the host were carried out with 2.0 mM host solutions. Stock solutions of NaOH were prepared at 286.0 mM. An aliquot of 0.5 mL of host was titrated in an NMR tube with careful addition of small aliquots of NaOH. Analysis of **1** with CsCl was performed by the addition of a 2.00 M CsCl solution to a 2.0 mM host solution such that the final CsCl concentration was 100 mM (50 equiv) and dilution of the host was 5% (Supporting Information File 1, Figure S3).

## Supporting Information

File 1 Additional analytical data and NMR spectra.
