# Supplementary material for: Dynamic light scattering studies of the effects of salts on the diffusivity of cationic and anionic cavitands
Source: Beilstein J Org Chem. 2018 Aug 23;14:2212–9. doi: 10.3762/bjoc.14.195 (PMC6122325; doi:10.3762/bjoc.14.195)
Supplement: File 1 — Additional analytical data and NMR spectra. [file Beilstein_J_Org_Chem-14-2212-s001.pdf]

Supporting Information

for

Dynamic light scattering studies of the effects of salts on the diffusivity of cationic and anionic cavitands

Anthony Wishard and Bruce C. Gibb*

Address: Department of Chemistry, Tulane University, New Orleans, LA 70118, USA

Email: Bruce C. Gibb - bgibb@tulane.edu

*Corresponding author

Additional analytical data and NMR spectra

Table of contents

Figure S1: PGSE NMR spectrum of **1** S2

Figure S2: ^{1}H NMR titration of **1** with NaOH S3

Figure S3: ^{1}H NMR of **1** with CsCl S4

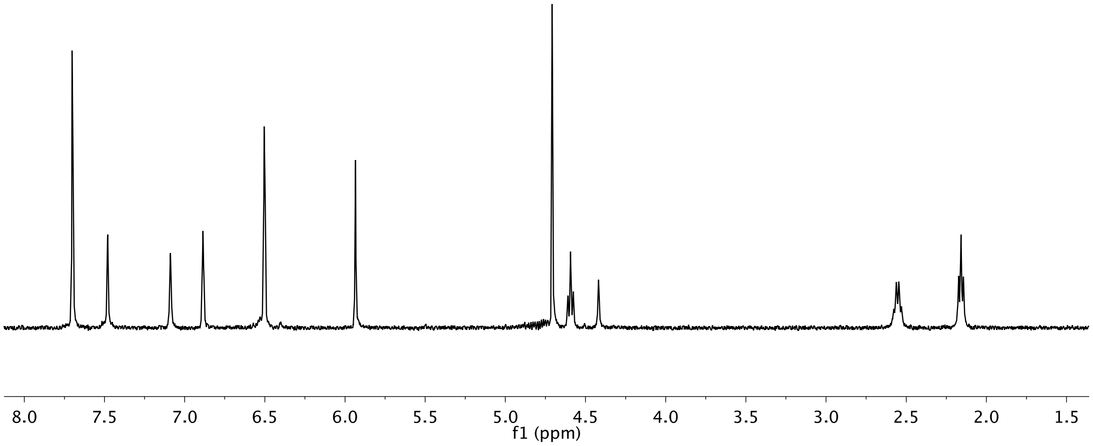

Figure S1: PGSE NMR of 2.00 mM **1** in 20.0 mM NaOH in H_2O.

Compound **1** requires a basic medium to facilitate solubility but is known to aggregate under mildly basic conditions, requiring an increase in pH to ensure monodispersity of the sample. Before performing DLS titrations, a ^{1}H NMR titration of **1** with NaOH was performed (Figure S2) to determine a suitable range where **1** would be monodisperse but the presence of excess salt would be minimized. The titration revealed that at 10 equiv (20 mM) NaOH (NMR 8 in Figure S2), the host is both fully dissolved and monodisperse. Cations play a muted role in the DLS measurements of this study and that fact, coupled with the highly solvated carboxylates of **1** (see main text) suggest that the presence of additional sodium ions should have a negligible impact on the results. Likewise, the hydroxide was mostly consumed in the deprotonation and solubilization of **1**, leaving only trace amounts present in solution. Again, this should have a negligible impact on the DLS results.

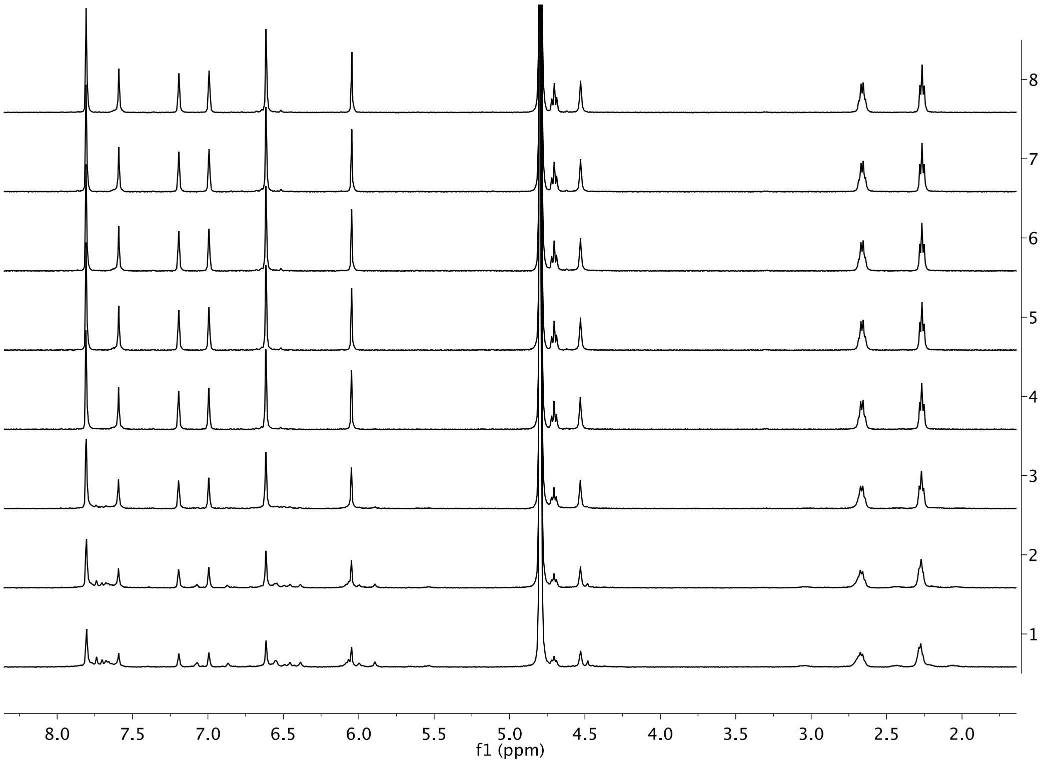

Figure S2: ^{1}H NMR titration of **1** with 268.0 mM NaOH from 6.5 (NMR 1) to 10 equiv (NMR 8).

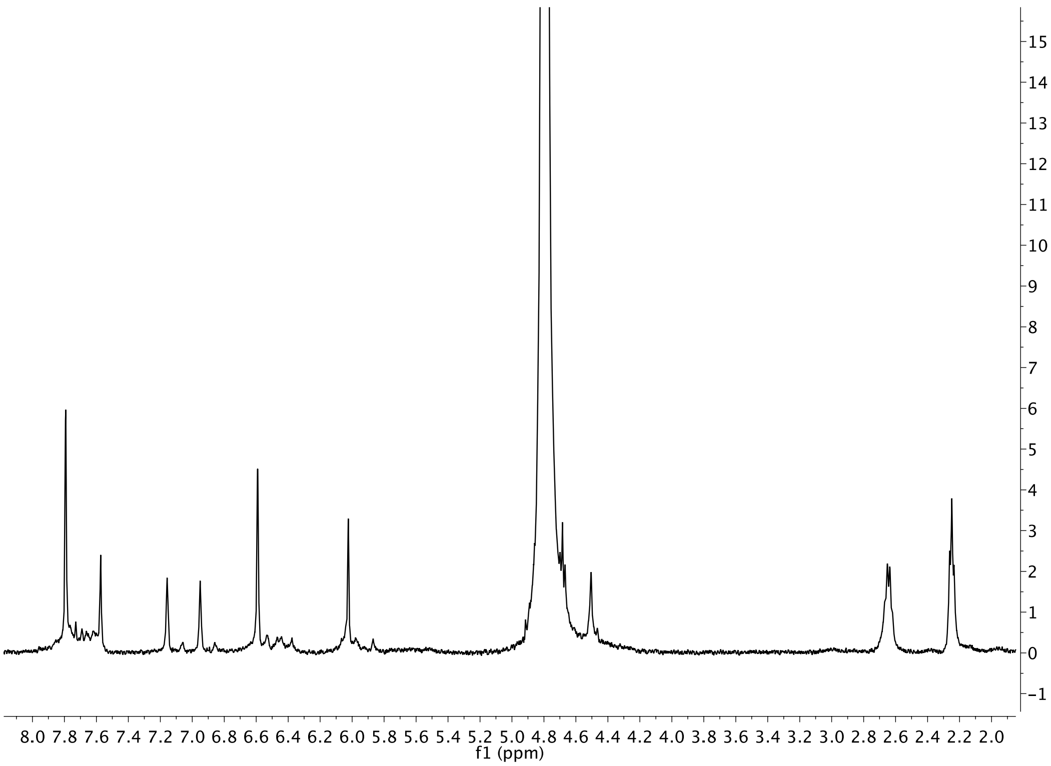

Figure S3: ^{1}H NMR of 2.0 mM **1** in 20 mM NaOH + 100 mM CsCl.
